# Measurement of geometric dephasing using a superconducting qubit

**DOI:** 10.1038/ncomms9757

**Published:** 2015-10-30

**Authors:** S. Berger, M. Pechal, P. Kurpiers, A. A. Abdumalikov, C. Eichler, J. A. Mlynek, A. Shnirman, Yuval Gefen, A. Wallraff, S. Filipp

**Affiliations:** 1 Department of Physics, ETH Zurich, CH-8093 Zurich, Switzerland; 2 Institut für Theorie der Kondensierten Materie, Karlsruhe Institute of Technology, 76128 Karlsruhe, Germany; 3 Department of Condensed Matter Physics, The Weizmann Institute of Science, Rehovot 76100, Israel

## Abstract

A quantum system interacting with its environment is subject to dephasing, which ultimately destroys the information it holds. Here we use a superconducting qubit to experimentally show that this dephasing has both dynamic and geometric origins. It is found that geometric dephasing, which is present even in the adiabatic limit and when no geometric phase is acquired, can either reduce or restore coherence depending on the orientation of the path the qubit traces out in its projective Hilbert space. It accompanies the evolution of any system in Hilbert space subjected to noise.

## Abstract (web summary)

Open quantum systems are subject to dephasing that ultimately destroys the information they hold. Here, the authors use a superconducting qubit to show that dephasing also has a geometric origin, which can either reduce or restore coherence depending on the path of the quantum system in its Hilbert space.

## Graphical abstract

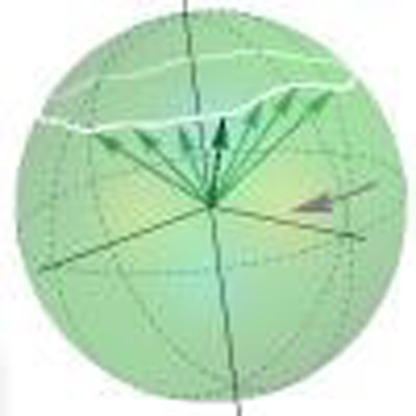

The information stored in a quantum bit is ultimately lost when the interaction with its environment causes randomization of its quantum phase in a process known as dephasing^1^. Part of this dephasing is of geometric origin^2^ and is related to a type of geometric phase known as Berry phase^3^,^4^, which is accumulated when a quantum system is adiabatically steered along a closed contour in the parameter space of its Hamiltonian. This phase underlines the dynamics and thermodynamics of a broad spectrum of quantum systems as measured in spins^5^,^6^, systems dominated by spin–orbit coupling^7^,^8^,^9^, experiments exhibiting Aharonov–Bohm phases^10^, as well as atomic^11^,^12^ and optical set-ups^13^,^14^.

The geometric phase roots in the structure of Hilbert space and is—unlike the dynamic phase—not related to the duration of a quantum process. When it comes to quantum dissipative systems, ubiquitous in our physical world, the nature of the geometric phase may be put to question: noise may screen out geometric effects and the condition for adiabaticity is not self-evident. The former effect is frequently modelled by adding non-geometric dissipative rates to the equations of motion for the density matrix, see for example, ref. 15. The latter concern is alleviated in ref. 16: naively one may expect that adiabaticity implies that the rate of change involved is smaller than the excitation gap. It turns out though that a gapless spectrum, which may be the result of coupling the system to a dissipative reservoir, does not necessarily annul the Berry phase^3^,^4^. Moreover, dephasing and level broadening due to classical low-frequency noise randomizing the Berry phase are addressed in refs 17, 18; corrections to the Berry phase, due to both slow, classical noise and high-frequency quantum noise have been discussed in ref. 2. In that work, the concept of geometric dephasing has been introduced. Indeed, decoherence in quantum systems stems not only from the stochastic evolution of the dynamic phase of the system's wave function (dynamic dephasing), but also from geometric effects.

In this article, we experimentally confirm the existence of geometric dephasing using a superconducting quantum system^19^ exposed to artificial noise. We find that geometric dephasing can either reduce or restore coherence depending on the orientation of the path a qubit traces out in its projective Hilbert space. This asymmetric decoherence mechanism is expected to play a role in numerous stochastic systems exhibiting geometric phases^20^,^21^.

## Results

### Experimental background

Our experiment complements earlier ones on orientation-independent dephasing induced by non-adiabatic corrections to the Berry phase^22^,^23^, and on the effect of classical noise^24^ and a quantum bath^25^ on the geometric phase.

Below, we show that the state (Bloch) vector of a qubit evolving adiabatically in the presence of Gaussian noise contains a suppression factor


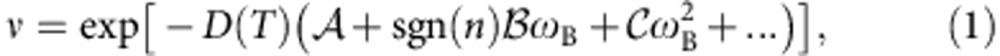


where 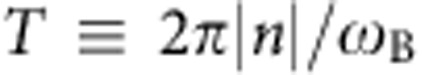 is the duration of the time evolution, 
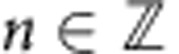
 the oriented number of loops of the qubit in its projective Hilbert space (equal to the number of loops performed by the magnetic field for adiabatic evolution), *ω*_B_>0 the precession frequency and 2*π*/*ω*_B_ the duration of a single loop. The function *D*(*T*) describes the spectral properties of the noise. The first term in equation (1) is independent of *ω*_B_ and represents dynamic dephasing. The second term, proportional to sgn(*n*)*ω*_B_, represents geometric dephasing. The third term goes as 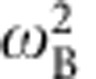 and stands for non-geometric non-adiabatic dephasing. It is especially noteworthy that the second term can either increase or decrease the total dephasing, depending on the sign of *n*. This makes the geometric nature of this term explicit. The contribution to dephasing stemming from the first term is independent of the sign of *n* and is therefore not geometric. Likewise, the third term does not describe geometric dephasing. However, it leads to fluctuations in the Berry phase^18^ as observed in refs 22, 23.

**Figure 1:**
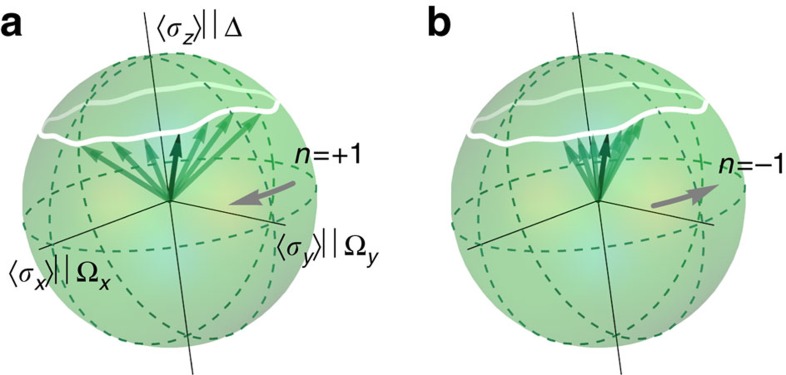
Qubit in a noisy environment. Bloch vectors (green arrows) describing the qubit state after evolving adiabatically in an precessing noisy magnetic field. When the qubit state evolves adiabatically, its Hilbert space can be identified with the parameter space of the Hamiltonian, that is, the three-dimensional effective magnetic field. The magnetic field (white line) follows a closed loop *n* times. In **a**, the number of loops is *n*=+1, in **b** it is *n*=−1. Owing to geometric dephasing, the Bloch vectors are fanned out more in **a** than in **b**.

**Figure 2:**
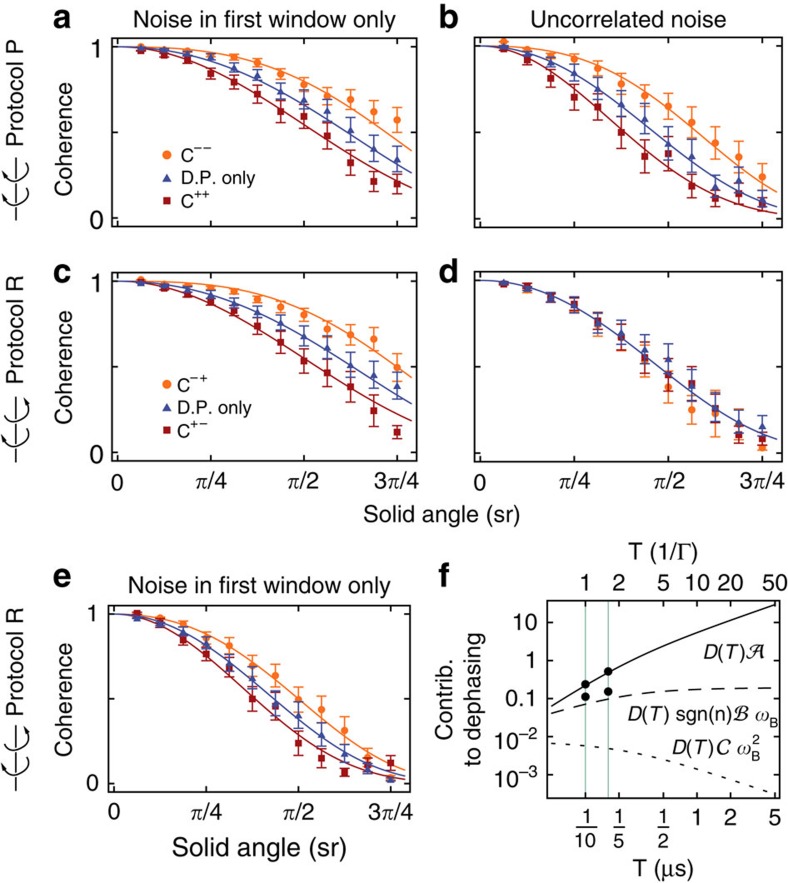
Coherence of the qubit. Measured normalized coherences as a function of solid angle with fluctuations in the effective magnetic field. Each data point is the average of 400 realizations of noise. If not stated otherwise, the precession period of the field is *T*=100 ns. Solid lines indicate fits to the data. The error bars indicate the s.d. (**a**) Coherence measured in a complete spin echo experiment (‘protocol P', see Fig. 3) with noise during the first window of the spin echo only. The direction of precession is preserved in the second window of the echo, leading to pulse sequences *C*^++^ and *C*^−−^. The exponents denote sgn(*n*), the direction of precession of the field. ‘dynamic phase (D. P.) only' denotes a magnetic field which does not precess (*ω*_B_=0), so that the qubit acquires only dynamic phase. (**b**) As in **a** but with uncorrelated noise. (**c**) Coherence measured in a spin echo in which the direction of precession of the magnetic field is reversed in the second half (‘protocol R'), giving *C*^+−^ and *C*^−+^. (**d**) As in **c** but with uncorrelated noise. (**e**) As in **c** but with precession period *T*=160 ns. (**f**) Dynamic (solid line), geometric (dashed line) and non-geometric non-adiabatic (dotted line) contributions to dephasing, as they appear in the exponent of *ν* in equation (1), as a function of precession period *T*. The curves are computed with the parameters used for recording the data shown in **c** and **e** for *A*=*π*/2. The values for dynamic and geometric dephasing extracted from fits to these measurements are indicated by black dots. The vertical lines indicate the periods *T*=0.1, 0.16 μs used in our experiments.

In our experiments, a superconducting qubit (Supplementary Methods and Supplementary Fig. 1) is subject to resonant driving with slowly modulated amplitude and phase. In a frame corotating with the drive, the system can be modelled as a qubit in a slowly varying magnetic field—a paradigmatic system to observe the Berry phase^5^,^22^,^26^. To study geometric dephasing, we add artificial noise to the drive, mimicking noise in the magnetic field. Our analysis shows explicitly that the decay of a pure quantum state into a mixed state includes a term that is exponential in *n*, the number of oriented loops: the dephasing is asymmetric in the direction of the loops, as schematically represented in Fig. 1. The coherence (that is, the length of the Bloch vector) of the qubit recorded in a Ramsey-type interferometric experiment illustrates this phenomenon. As shown in Fig. 2a, a cyclic change of the effective magnetic field in clockwise (*C*^++^) or counter-clockwise (*C*^−−^) direction leads either to a decrease or an increase in coherence when compared with a static but still fluctuating magnetic field, as discussed in detail below.

The dynamics of the system in a frame rotating at the frequency *ω*_d_ of the drive are described by the Hamiltonian (ℏ=1)^26^


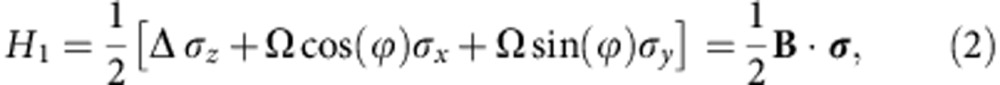


where **σ**=(*σ*_*x*_, *σ*_*y*_, *σ*_*z*_) are the Pauli matrices, Δ≡*ω*_01_−*ω*_d_ the detuning in frequency between drive and qubit transition, Ω(*t*) the amplitude and 
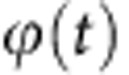
 the phase of the drive and 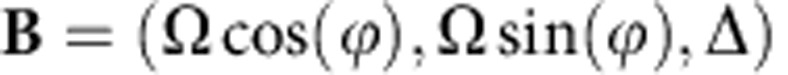 the effective magnetic field in units of angular frequency. We let the magnetic field form an angle *θ*=arctan Ω/Δ with the Δ axis and have it precess about this axis at a rate 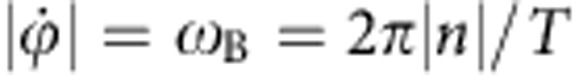. After a time *T*, the magnetic field has traced out *n* loops and enclosed a solid angle *A*=2*nπ*(1−cos*θ*) as seen from **B**=0. If *ω*_B_ is small enough, that is, if the evolution is adiabatic, the qubit's state vector acquires a geometric phase *A*/2 (ref. 4). We induce fluctuations *δ*Ω in the effective magnetic field in radial direction, which in our set-up correspond to amplitude noise in the signal driving the qubit, so that Ω(*t*)=Ω_0_+*δ*Ω(*t*) and as a consequence *θ*(*t*)=*θ*_0_+*δθ*(*t*). Applying a transformation 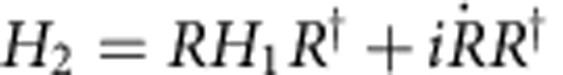 with 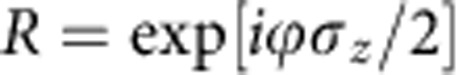 (see for example, ref. 27) to equation (2) results in the Hamiltonian


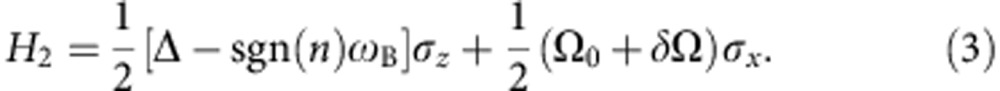


From equation (3), it follows that in a Ramsey experiment, in which the effective magnetic field performs *n* oriented loops in the time interval [0, *T*], the eigenstates of the qubit acquire a total relative phase *γ*([0, *T*], *n*), where


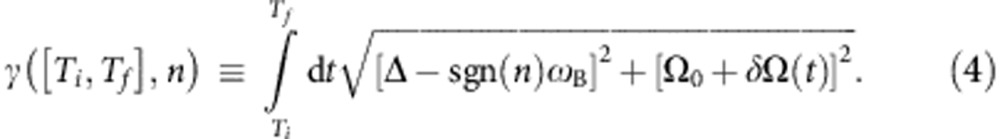


We make sure that the conditions 
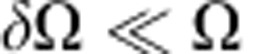
 and 
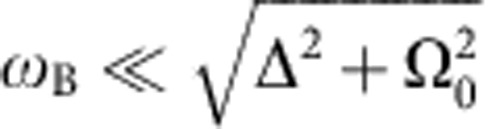
 are met, so that the qubit evolves adiabatically (also see Methods section). A second-order Taylor expansion of equation (4) in the two small parameters *δ*Ω and *ω*_B_ yields


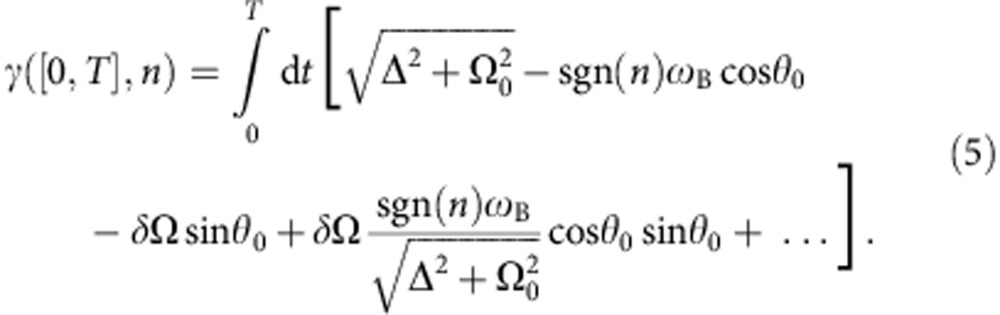


In this expansion, where *θ*_0_ ≡ arctanΩ_0_/Δ, we have dropped two second-order terms which are non-consequential for our analysis: one 
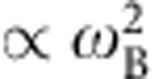
 (non-adiabatic corrections) and one ∝*δ*Ω^2^ (next-order correction to the dynamical phase and to the dynamical dephasing). The first two terms in equation (5) give rise to 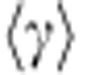, the sum of dynamic and geometric phase in the absence of noise. The last two terms lead to dephasing, as seen by computing the variance 
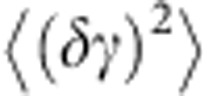
 of the phase in equation (5),


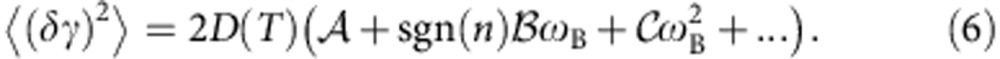


Thus, we recover the suppression factor from equation (1) describing the length 
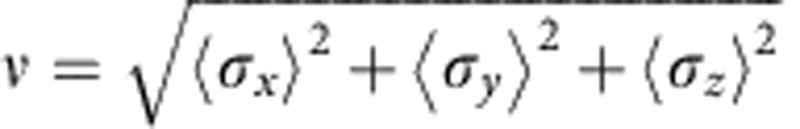
 of the state vector of the qubit. The function


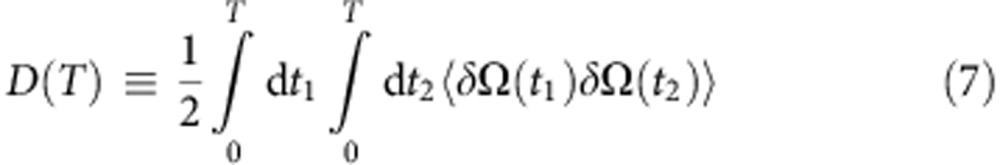


is the integrated time-correlator of the noise. The functions


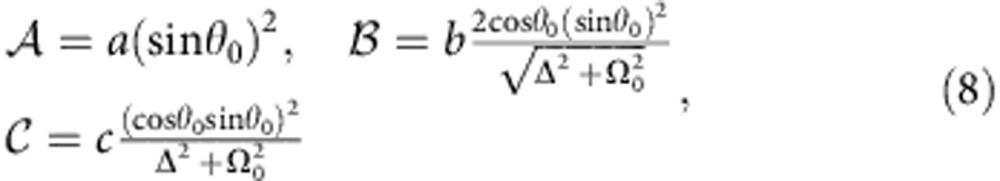


appear in the three first terms in equation (6), which represent dynamic dephasing, geometric dephasing and non-geometric non-adiabatic corrections, respectively.

In equation (8) we have introduced dimensionless decoherence factors *a*, *b*, *c* (all equal to one in the Ramsey experiment considered) to accommodate different types of experiments, as detailed below. In particular, we use spin echo techniques to observe geometric dephasing while eliminating the dynamic phase and enhancing the qubit coherence time.

### Protocols for measuring dephasing

Two protocols leading to different decoherence factors *a*, *b*, *c* are considered. The first one is a complete echo, in which the orientation of the loop the magnetic field traverses is identical in both halves. In this protocol (named ‘protocol P', as in ‘preserved'), the phase acquired by the qubit is


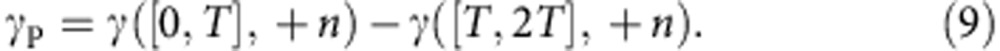


In the second protocol, the orientation of the loop is reversed in the second half of the echo sequence. We therefore call it ‘protocol R' (for ‘reversed'). The accumulated phase is


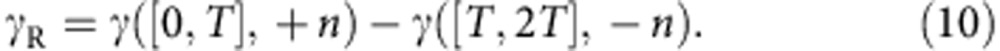


**Figure 3:**
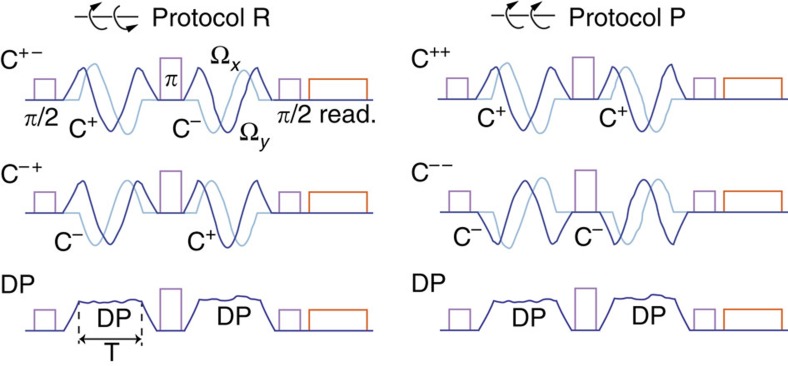
Pulse sequences. Pulse sequences for protocol R (where the direction of precession of the magnetic field is reversed in the second half of the spin echo) and protocol P (where it is preserved). The *π*- and *π*/2-pulses implementing the spin echo are on resonance with the qubit transition frequency *ω*_01_ (purple). At the end of the sequence, the state of the qubit is read out by applying a tone at frequency *ω*_r_ (orange). The components Ω_*x*_ and Ω_*y*_ of the magnetic field are shown in dark and light blue. Δ is kept constant and is not shown.

To illustrate the protocols, schematics of the pulse sequences are shown in Fig. 3.

In our experiment, phase and coherence of the qubit have been recorded as a function of the solid angle enclosed by the effective magnetic field **B**. The detuning of the off-resonant pulses is Δ/2*π*=−35 MHz. The fluctuations *δ*Ω applied to **B** conform to an Ornstein–Uhlenbeck process with correlation time 1/Γ=10 MHz, intensity *σ*^2^ and normalized noise amplitude 
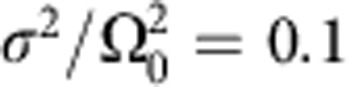
. Intrinsic dephasing due to a finite decoherence rate of the qubit causes the coherence to drop to about 0.7 at the end of the pulse sequence. This effect is calibrated out^23^. The correlation function of the noise process is 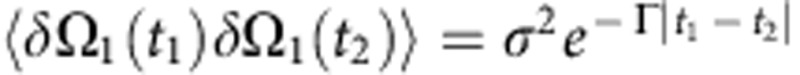, and the integrated time-correlator from equation (7) is 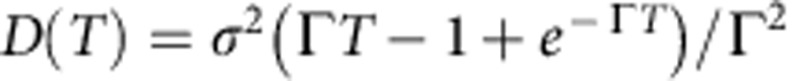. Since the noise *δ*Ω(*t*) is artificial, we may control its time correlations, in particular those between the first time window, 0<*t*<*T* and the second time window, *T*<*t*<2*T*. For convenience, we define *δ*Ω_1_(*t*) ≡ *δ*Ω(*t*) and *δ*Ω_2_(*t*) ≡ *δ*Ω(*t*+*T*).

### Geometric and dynamic dephasing

A spin echo sequence with noise in the first window only, that is, *δ*Ω_2_(*t*)≡0, allows us to measure the dephasing the qubit experiences during a Ramsey experiment. There is geometric dephasing in both protocols (*b*≠0 in Fig. 2a,c,e) which, depending on the sign of *n*, either increases or reduces the total dephasing. Interestingly, in protocol P geometric dephasing is present although the Berry phase (not shown) is eliminated along with the dynamic phase. This phenomenon can be explained by computing the variance of the phases in equations (9) and (10), from which the decoherence factors *a*=1, *b*=1 follow. In our experiment, a fit to the data yields a=1.14±0.02 for protocol P and *a*=1.07±0.02 for protocol R (Table 1), in good agreement with computations. The observed geometric dephasing (*b*=1.46±0.05 for protocol P and 1.58±0.12 for protocol R) is somewhat larger than predicted. The procedure used to extract *a* and *b* from fits to the data is described in the Methods section. The measured phases in protocol R agree with the prediction for a weakly anharmonic multi-level system^28^, with and without applied noise. However, when the noise causes the coherence to drop below ≈0.2, the phase can no longer be determined reliably and the measured phase deviates from theory.

Dynamic dephasing is more pronounced if the sequence is longer (*T*=160 ns in panel e, versus *T*=100 ns in panel c), but geometric dephasing is always present. The time-dependence in equation (1) describes the experimental data in Fig. 2 well, with *a*=1.12±0.03 and *b*=1.45±0.15 (for *T*=160 ns) and *a*=1.14±0.02 and *b*=1.58±0.12 (for *T*=100 ns). Examining the three contributions to dephasing appearing in the coherence suppression factor in equation (1) as a function of *T* (Fig. 2f), we see that the dynamic contribution 
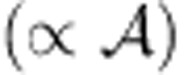
 grows with *T*, while the geometric contribution 
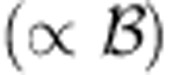
 saturates in the limit of short correlation times 
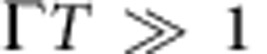
: it does not vanish in the adiabatic limit *T*→∞. The non-geometric non-adiabatic contribution 
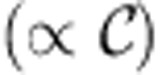
 vanishes in this limit. This holds for a general noise process (see Supplementary Methods).

While the experiment with noise in the first half of the spin echo only is adequate to measure geometric dephasing, a more physically relevant scenario is to consider noise appearing in both halves of the spin echo.

For uncorrelated noise, that is, when the correlation time of the noise is shorter than the timescale of the spin echo (as for example, for white noise^29^,^30^), we have 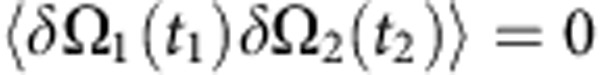. In addition, the spectral power of the noise is kept constant, 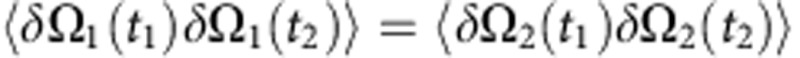. In this case, we measure geometric dephasing in the complete spin echo only (protocol P, Fig. 2b and Table 1). In protocol R, there is only dynamic dephasing (Fig. 2d, see Methods section for details).

For perfectly correlated noise *δ*Ω_1_(*t*)=*δ*Ω_2_(*t*), which corresponds to fluctuations slower than typical spin echo time scales as typical for 1/*f*-noise, neither dynamic nor geometric dephasing is expected. Non-adiabatic contributions (quantified by *c*) only play a role in protocol R, where *c*=4 is expected and *c*=2.25±0.25 is found from a fit to data (not shown). In protocol P, all fluctuations cancel out (*a*=*b*=*c*=0 in theory) and a fit gives *c*=0.24±0.54. The effect of correlated noise has been studied in refs 18, 22, 23.

Finally, we have also considered anticorrelated noise (*δ*Ω_1_(*t*)=−*δ*Ω_2_(*t*)) which maximizes both dynamic and geometric dephasing (Table 1) in accordance with expectations. As with uncorrelated noise, geometric dephasing is only present in protocol P.

Although in this experiment we consider geometric dephasing for closed loops, this effect may be detected even for open trajectories in parameter space: In any interferometric set-up, interference fringes originating from the non-unity overlap between the initial reference state and the final state after a non-cyclic evolution will exhibit dephasing in the form of a decaying envelope. Geometric dephasing will reveal itself as a rate of decay which depends on the direction, that is, the sign of *n*.

Given the broad spectrum of systems whose dynamics involves geometric phases, the presence of geometric dephasing is expected to be commonplace. While this additional contribution to decoherence is typically small, it can be of relevance for high-fidelity quantum operations and decoupling pulse techniques. For example, residual coupling to spurious modes will cause the system of interest to precess and induce dephasing with both dynamical and geometric contributions. Moreover, as shown here, geometric dephasing is present even when no geometric phase is acquired. One resulting intriguing question concerns the effect of geometric dephasing on the braiding phase of topological quasiparticles, when the separation of the relevant particles is within reach of stochastic fluctuations of the braiding path.

## Methods

### Considerations about adiabaticity

The adiabaticity parameter is defined according to ref. 31. Given a Hamiltonian *H*(*t*), we can define an instantaneous (adiabatic) basis such that 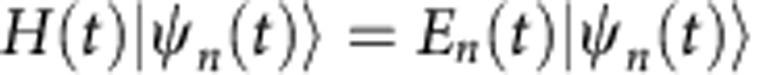. Writing *D*(*t*) for the transformation from a fixed basis (given for example, by H(0)) to the instantaneous basis, the Hamiltonian in the instantaneous basis is 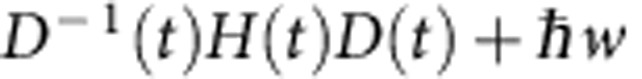, with 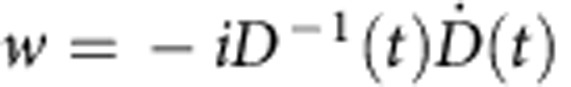. In the adiabatic case, *w* vanishes. The adiabatic parameter is defined as 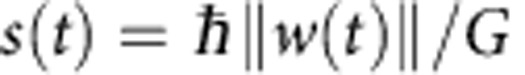, where 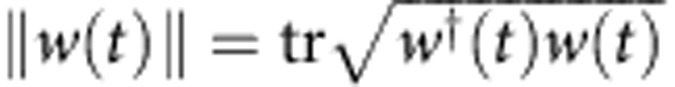 is the trace norm of *w*(*t*) and 
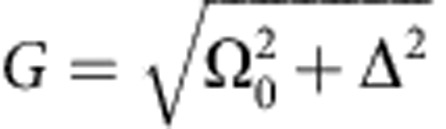
 is the energy gap in the spectrum of *H*(0). Evolution is adiabatic if 
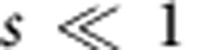
.

**Figure 4:**
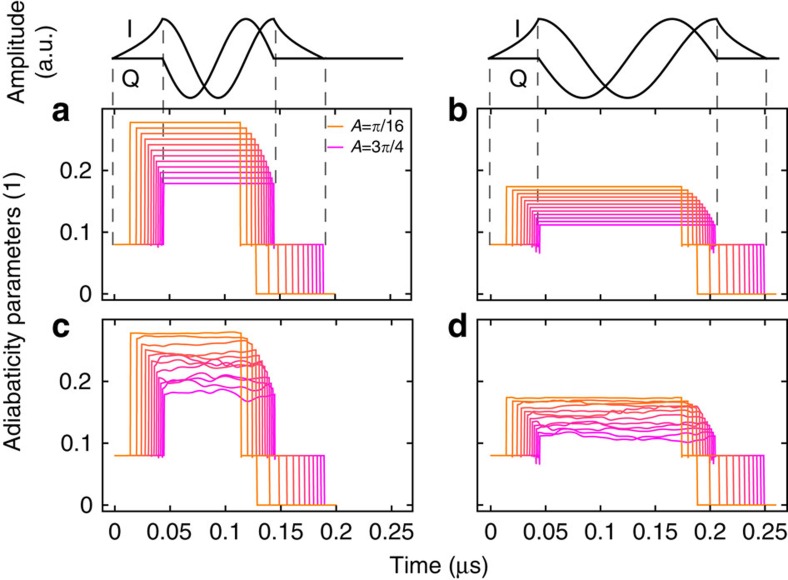
Adiabaticity parameter. Adiabaticity parameter during the off-resonant part of the pulse sequence for *ω*_B_/2*π*=*T*=100 ns (**a**) and 160 ns (**b**) without applied noise. Graphs for an example of a pulse sequence with applied noise are shown in panels **c** and **d**. In each panel, the adiabaticity parameter is shown for solid angles *A*=*π*/16 (orange) to 3*π*/4 (pink) in steps of *π*/16. The pulse envelopes for *A*=3*π*/4 are shown on top of the plots. Note that the curves are not offset in time; the pulses have different lengths.

The off-resonant pulses are shaped such that *s* is constant over time and independent of the solid angle when the drive Ω is increased or decreased (that is, at beginning and end of the pulses). For larger solid angles, the pulses need to be longer to keep *s* constant across solid angles. When the effective magnetic field precesses (central part of the pulses), *s* varies from solid angle to solid angle because *ω*_B_ is kept constant. The data in Fig. 4 shows that *s*(*t*) is always smaller than 0.28 and thus adiabaticity is maintained during the whole off-resonant pulse sequence, even when noise is applied. As expected, *s* is smaller for 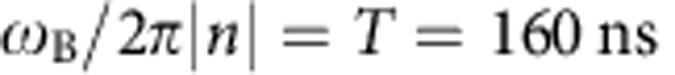 than for *T*=100 ns.

Going to the extreme adiabatic limit *s*→0 is not desirable. Although geometric dephasing is still present in this limit, it cannot be resolved experimentally because dynamic dephasing increases linearly with time (see Supplementary Methods).

### Fitting the measured coherences

This section describes the fit models used to extract the parameters *a*, *b* and *c* quantifying dynamic and geometric dephasing.

In the fitting procedure, in a first step the effective normalized noise amplitude is found by fitting the function given in equation (1) describing the coherence *ν* to the data from protocol R with noise in the first window and dynamic phase only, assuming *a*=1, *b*=0, *c*=0 and with the normalized noise amplitude as the only fit parameter. In this way, a normalized noise amplitude of 
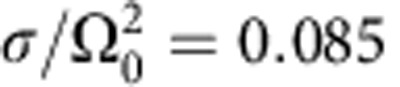
 was determined, which is slightly smaller than the set value 0.1.

In a second step, all data from protocol R are fitted simultaneously for coefficients *a* and *b* of the functions 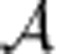 and 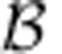. Where the theory predicts *b*=0, we do not wish to constrain the fitting function (and, by extension, limit the model) by setting *b*=0. Rather, we use a fitting function with a separate, primed variable, which ideally the fit then shows to be zero. We note that using the same variable *b* as in the other fits, where *b*≠0 is expected, is not possible, since all data is fitted simultaneously. As an example, consider protocol R with anticorrelated noise and *C*^−+^, where according to Table 2 no geometric dephasing (*b*=0) is expected: a fitting function with *b*→*b*′ is used and the fit ideally produces *b*′=0. Similarly, for protocol R, dynamic phase, a variable *b*′′ is introduced to avoid unwanted interference with *b*′, as using a single parameter would prevent *b*′ from taking values other than zero in the fits to protocol R, *C*^+−^ and *C*^−+^ , where *b*′=0 is expected. Finally, the proportionality factors of *a* and *b* in the fitting functions are fixed across the measurements. For instance, protocol R with anticorrelated noise uses 4*a* and protocol R, and with uncorrelated noise uses 2*a*. The data from protocol P is fitted similarly.

In a third step, the parameter *c* is only fitted for protocol R, correlated noise, *C*^±^, where *a* and *b* vanish. This is the only measurement where it is relevant.

Fourth, the fitting parameters *b*′ and *b*′′ are set to zero in the fit we call ‘constrained'. When dropping this constraint, the fitted values for *a* and *b* increase slightly (by maximally 6%, see Table 3); in turn, *b*′ and *b*′′ become negative. Simply put, the ‘unconstrained' model (where *b*′ and *b*′′ are free parameters) trades-off some fitting parameters against each other to obtain the best fit. If theory was in perfect agreement with experimental data (no noise, no systematic errors), this trade-off would not be possible. However, owing to experimental imperfections the fit produces an unphysical result, namely ‘negative' dynamic dephasing (*b*′, *b*′′<0). To avoid this effect, we have opted for presenting the constrained model in the main text. Comparing the parameter estimates in Table 3, it becomes apparent that both models yield similar parameter estimates. In addition, as discussed in the Supplementary Methods and in Supplementary Fig. 2, both models (constrained and unconstrained) have empirical support.

## Additional information

**How to cite this article:** Berger, S. *et al.* Measurement of geometric dephasing using a superconducting qubit. *Nat. Commun.* 6:8757 doi: 10.1038/ncomms9757 (2015).

## Supplementary Material

Supplementary Information Supplementary Figures 1-2, Supplementary Methods and Supplementary References

## Figures and Tables

**Table 1:** Decoherence factors.

**Correlation of noise**	**Theory**						**Experiment**			
	**Protocol R**			**Protocol P**			**Protocol R**		**Protocol P**	
	***a***	***b***	***c***	***a***	***b***	***c***	***a***	***b***	***a***	***b***
Correlated, *δ*Ω_1_=*δ*Ω_2_	0	0	4	0	0	0	—	—	—	—
Anticorrelated, *δ*Ω_1_=−*δ*Ω_2_	4	0	0	4	4	4	4.29±0.07	—	4.54±0.06	5.84±0.20
Uncorrelated, 〈*δ*Ω_1_*δ*Ω_2_〉=0	2	0	2	2	2	2	2.14±0.04	—	2.27±0.03	2.92±0.10
First window, *δ*Ω_2_=0	1	1	1	1	1	1	1.07±0.02	1.58±0.12	1.14±0.02	1.46±0.05

Decoherence factors for spin echo experiments with noise on the effective magnetic field. Two different protocols are considered, with four types of noise correlations between the first and the second halves of the echo sequence (cf. text). Theoretical decoherence factors (see equation (8)) associated with dynamic dephasing (*a*), geometric dephasing (*b*) and non-geometric corrections originating from the stochasticity of the Berry phase (*c*).

**Table 2:** Fitting functions for quantifying dephasing.

	**Unconstrained fit**						**Constrained fit**					
	**Protocol R**			**Protocol P**			**Protocol R**			**Protocol P**		
	***C***^**+−**^	**DP**	***C***^**−+**^	***C***^**++**^	**DP**	***C***^**−−**^	***C***^**+−**^	**DP**	***C***^**−+**^	***C***^**++**^	**DP**	***C***^**−−**^
C	(0, −*b*′, 4*c*)	(0, *b*′′, 0)	(0, *b*′, 4*c*′)	(0, *b*′, *c*′)	(0, *b*′, *c*′)	(0, *b*′, *c*′)	(0, 0, 4*c*)	(0, 0, 0)	(0, 0, 4*c*)	(0, 0, *c*′)	(0, 0, *c*′)	(0, 0, *c*′)
A	(4*a*, −*b*′, 0)	(4*a*, *b*′′, 0)	(4*a*, *b*′, 0)	(4*a*, −4*b*, 0)	(4*a*, *b*′, 0)	(4*a*, 4*b*, 0)	(4*a*, 0, 0)	(4*a*, 0, 0)	(4*a*, 0, 0)	(4*a*, −4*b*, 0)	(4*a*, 0, 0)	(4*a*, 4*b*, 0)
U	(2*a*, −*b*′, 0)	(2*a*, *b*′′, 0)	(2*a*, *b*′, 0)	(2*a*, −2*b*, 0)	(2*a*, *b*′, 0)	(2*a*, 2*b*, 0)	(2*a*, 0, 0)	(2*a*, 0, 0)	(2*a*, 0, 0)	(2*a*, −2*b*,)	(2*a*, 0, 0)	(2*a*, 2*b*, 0)
1	(*a*, −*b*, 0)	(*a*, *b*′, 0)	(*a*, *b*, 0)	(*a*, −*b*, 0)	(*a*, *b*′, 0)	(*a*, *b*, 0)	(*a*, −*b*, 0)	(*a*, 0, 0)	(*a*, *b*, 0)	(*a*, −*b*, 0)	(a, 0, 0)	(*a*, *b*, 0)

DP, dynamic phase.

Fitting functions used to obtain the fit estimates for *a* and *b* presented in Table 3. The column headings are abbreviations for the correlations of the noise, viz. correlated (C), anticorrelated (A), uncorrelated (U) and noise in the first window (1). Reading example: when the fit is unconstrained, the coherences for protocol R with correlated noise and *C*^+−^ are fitted using the function *ν* from equation (1) with coefficients *a*→0, *b*→−*b*′ and *c*→4*c*.

**Table 3:** Parameter estimates quantifying dephasing.

**Correlation of noise**	**Unconstrained fit**				**Constrained fit**			
	**Protocol R**		**Protocol P**		**Protocol R**		**Protocol P**	
	***a***	***b***, ***b*****′**	***a***	***b***, ***b*****′**	***a***	***b***	***a***	***b***
Correlated	—	0.06±0.07	—	−0.30±0.12	—	—	—	—
Anticorrelated	4.31±0.08	0.06±0.07	4.63±0.07	6.00±0.21	4.29±0.07	—	4.54±0.06	5.84±0.20
Uncorrelated	2.15±0.04	0.06±0.07	2.32±0.04	3.00±0.11	2.14±0.04	—	2.27±0.03	2.92±0.10
First window	1.08±0.02	1.59±0.12	1.16±0.02	1.50±0.05	1.07±0.02	1.58±0.12	1.14±0.02	1.46±0.05

Parameters estimates for *a* and *b* (respectively *b*′) extracted from constrained and unconstrained fits to data in Fig. 2 and additional data (not shown). For the unconstrained fit to the data of protocol R with dynamic phase, *b*′′=−0.08±0.08 is found.
