# Supplementary material for: Measurement of geometric dephasing using a superconducting qubit
Source: Nat Commun. 2015 Oct 30;6:8757. doi: 10.1038/ncomms9757 (PMC4640075; doi:10.1038/ncomms9757)
Supplement: Supplementary Information — Supplementary Figures 1-2, Supplementary Methods and Supplementary References [file ncomms9757-s1.pdf]

SUPPLEMENTARY FIGURE 1

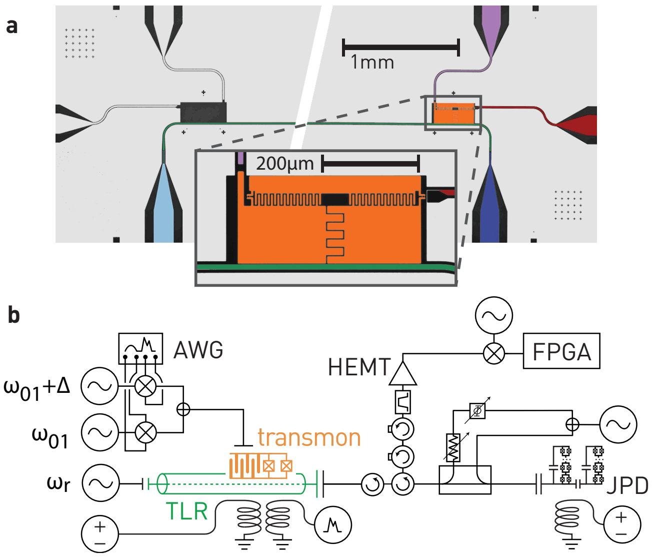

Supplementary Figure 1. (a) Micrograph of the sample. Resonator input (dark blue), resonator output (light blue), resonator (green), charge line (red), flux line (violet) and transmon qubit (orange), and zoom on the qubit (inset). (b) Schematic of the measurement setup. See text for details.

SUPPLEMENTARY FIGURE 2

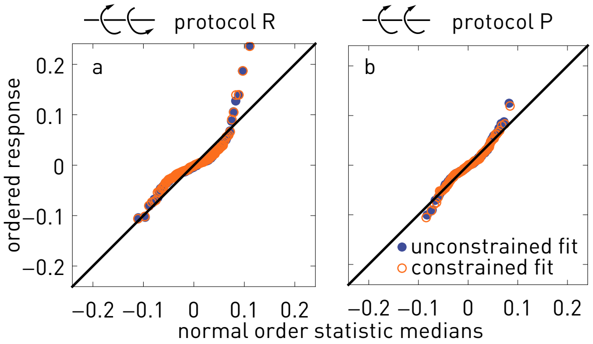

Supplementary Figure 2. Normal probability plots of the residues of the fits from protocol R and P. The line indicates what is expected for a perfect normal distribution.

SUPPLEMENTARY METHODS

Dependence of decoherence on the temporal correlations of the noise

In this appendix, an arbitrary noise process is considered, characterized by its intensity σ^2 (the integral of its power spectral density) and its correlation time $1/\Gamma$. We show that only the second term $\propto (\omega_B)^1$ of Eq. (1) in the main manuscript represents geometric dephasing by estimating the coherence suppression factor ν in two limiting cases: noise with short correlation time ($\Gamma T \gg 1$) and noise with long correlation time ($\Gamma T \ll 1$).

Short correlation time. In this case, $\Gamma T \gg 1$ and therefore $D(T) \propto \sigma^2 T/\Gamma$. Eq. (1) in the main manuscript thus becomes

$$\nu = \exp\left[-O(1)\frac{2\pi\sigma^2}{\Gamma}\left(\frac{\mathcal{A}}{\omega_B}|n| + \mathcal{B}\,n + \mathcal{C}\omega_B\,|n| + ...\right)\right] \tag{1}$$

and we see that only the second term depends on the sign of n and represents geometric dephasing.

Long correlation time. Here $\Gamma T \ll 1$, and we obtain $D(T) \propto \sigma^2 T^2$, leading to

$$\nu = \exp\left[-O(1)(2\pi\sigma)^2\left(\frac{\mathcal{A}}{\omega_B^2}|n|^2 + \frac{\mathcal{B}}{\omega_B}|n|n + \mathcal{C}\,|n|^2 + ...\right)\right]. \tag{2}$$

Again, only the second term contributes to geometric dephasing. To sum up, there is a geometric contribution to dephasing regardless of the correlation time of the noise (or, put differently, regardless of the duration of the evolution).

Description of the sample and the setup

The sample (Supplementary Figure 1) is a superconducting circuit coupled to a transmission line resonator (TLR)[1]. The qubit consists of three superconducting islands (shown in orange in Supplementary Figure 1) and two squid loops. This device[2] can be seen as two coupled transmons forming a superconducting qubit with tunable frequency and tunable coupling to the resonator. Both parameters are tuned using a static bias current applied to a flux line and a miniature coil mounted below the sample. In the experiment presented here, the bright state[2] serves as a qubit, whose coupling is tuned to $g/2\pi = 38\,\mathrm{MHz}$ and kept constant. Its lowest transition is set to a frequency $\omega_{01} = 7.0335\,\mathrm{GHz}$. The dark state[2] is tuned so that its transition frequency lies above ω_{01} and thus it can safely be ignored.

The qubit has an anharmonicity of $\alpha/2\pi = 90\,\mathrm{MHz}$ as determined by spectroscopical measurements. It is inherently low due to the tunable-coupling design. When applying resonant pulses on the transition $|0\rangle \leftrightarrow |1\rangle$, a pulse-shaping technique known as derivative removal by adiabatic gate[3] is employed to avoid populating the higher excited states of the qubit.

The fundamental mode of the resonator is at a frequency $\omega_r/2\pi = 7.347\,\mathrm{GHz}$ and has a loaded quality factor of $Q = 3600$. We dispersively read out[4] the quantum state of the qubit by monitoring the transmission of a rf-signal at frequency ω_r trough the resonator. The input port of the resonator is coupled less strongly to the transmission line than the output port to increase the SNR of the readout.

Via two circulators and a directional coupler, the output signal of the resonator goes to an amplifier based on a Josephson parametric dimer (JPD, Ref. 5), with a gain of 18.4 dB at a bandwidth of 28 MHz centered around 7.348 GHz. The first circulator prevents reflected signals in the output line from leaking into the cavity; the second circulator separates the output of the JPD amplifier from the input. The directional coupler is used to operate the JPD amplifier: a tone is split, the first half pumps the JPD and the second is phase-shifted and attenuated so that it cancels the pump tone in the amplified signal. The pump tone is applied at a frequency $\omega_p = 7.564\,\mathrm{GHz}$, detuned 217 MHz from the resonator.

The signal is then bandpass filtered (4 to 8 GHz) and amplified with a high-electron-mobility transistor (HEMT) providing 35 dB of gain. At room temperature, the signal is amplified further, filtered and downconverted to 25 MHz before it is digitized at a rate of 100 MS/s with an analogue-to-digital converter and then processed with a field-programmable gate array (FPGA).

We use direct modulation of an in-phase/quadrature mixer to generate the microwave pulses for qubit state manipulation. The pulses are applied through a capacitively coupled charge bias line. The I and Q quadratures are synthesized with an arbitrary waveform generator (AWG). The pulses resonant with the qubit transition have a gaussian envelope with a standard deviation of $\sigma = 10\,\mathrm{ns}$. They are symmetrically truncated to a length of 4σ. Measuring Rabi oscillations allows us to extract the amplitudes of π- and $\pi/2$-pulses. We perform Ramsey interferometry experiments to calibrate the frequency of these pulses, which also serve to extract the dephasing time $T_2^\star = 770\,\mathrm{ns}$ and echo-decay-time $T_2^{\mathrm{echo}} = 1520\,\mathrm{ns}$ of the ω_{01}-transition. The lifetime of the first excited state is found to be $T_1 = 1330\,\mathrm{ns}$.

Comparison of fitting models

In this section, the constrained and the unconstrained fitting models are compared.

The normal probability plots[6] of the residues of the models, Supplementary Figure 2, show that the residues fall near the line describing the identity function, with the exception of a few residues which are larger than expected. Therefore, they are normally distributed. This holds for protocols R and P in both models (constrained and unconstrained). The underlying normal probability distributions of the residuals assume a mean 0 and use the standard deviation computed from the residuals:

$$\sigma^{(R)}_{\text{unc.}} = 0.0425, \quad \sigma^{(R)}_{\text{con.}} = 0.0427, \quad \sigma^{(P)}_{\text{unc.}} = 0.0319, \quad \sigma^{(P)}_{\text{con.}} = 0.0326. \tag{3}$$

To estimate if constrained and unconstrained models fit similarly well, we consider the Akaike information coefficient (AIC)[7]. The number of fitting parameters is

$$k^{(R)}_{\text{unc.}} = 6, \quad k^{(R)}_{\text{con.}} = 4, \quad k^{(P)}_{\text{unc.}} = 5, \quad k^{(P)}_{\text{con.}} = 4, \tag{4}$$

where we take into account the fact that the variance of the residuals Eq. (3) is estimated from the model. Since the number of fitting parameters k is not very small compared to the sample size $N = 3 \cdot 4 \cdot 12 = 144$ (the number of measured coherences), we use AIC_c, the finite-sample-corrected AIC. Because the residuals are normally distributed, and assuming the variance of the residuals is constant, AIC_c takes on the simple form

$$\text{AIC}_c = N \ln(\text{RSS}/N) + 2k + \frac{2k(k+1)}{N-k-1}, \tag{5}$$

with the residual sum of squares RSS. When comparing two models, the better model has the smaller AIC_c value. As a rough rule of thumb[7], if the difference in AIC_c with the other model lies between 0 and 2, that model has substantial empirical support, if between 4 and 7, it has less support, and if larger than 10 it should not be considered. Here, for protocol R the unconstrained model is the better model and the difference in AIC_c with the constrained model is 2.41. For protocol P, the converse is true: The constrained model is a better model and the difference in AIC_c with the unconstrained model is 4.31. To sum up, for both protocols the constrained model and the unconstrained one have some empirical support.

In addition, a t-test has been performed to assess the significance of the parameters a, b, b', c, c' in the unconstrained model. With a sample size of $N = 144$ and $k^{(R)}_{\text{unc.}} = 5$,

respectively $k^{(P)}_{\text{unc.}} = 4$ degrees of freedom, we find (for both) a threshold $t = 1.97$ at which a parameter value has a non-zero value with 95% of significance in a two-sided t-test. Given that

$$t^{(R)}_a = 55.53, \quad t^{(R)}_b = 12.79, \quad t^{(R)}_{b'} = 0.88, \quad t^{(R)}_c = 9.18, \tag{6}$$

and

$$t^{(P)}_a = 65.22, \quad t^{(P)}_b = 28.14, \quad t^{(P)}_{b'} = -2.56, \quad t^{(P)}_{c'} = 2.56, \tag{7}$$

it can be asserted that in both protocols the parameters a quantifying the dynamic dephasing and b quantifying the geometric dephasing are significant. Furthermore, the t-values for the parameter b' are close to zero (as it should be, since we expect $b' = 0$), indicating that in this measurement there is no geometric dephasing. Finally, the value $t^{(R)}_c$ indicates significance of the parameter c in protocol R, where non-geometric non-adiabatic dephasing is present. In protocol P, where $c' = 0$ is expected, it is only weakly significant.

We note that fitting for the parameters a, b and c for individual data sets with $n = 12$ (such as protocol R with correlated noise and C^{-+}) or groups of data sets with $n = 36$ (such as protocol R with correlated noise and either C^{-+}, C^{+-} or D.P.) does not produce useful parameter estimates. The same phenomenon as described above, the trading off of some geometric dephasing against dynamic dephasing, is exacerbated and the fit values are not significant.

SUPPLEMENTARY REFERENCES

[1] Koch, R. H., DiVincenzo, D. P. & Clarke, J. Model for $1/f$ flux noise in squids and qubits. *Phys. Rev. Lett.* **98**, 267003 (2007).

[2] Srinivasan, S. J., Hoffman, A. J., Gambetta, J. M. & Houck, A. A. Tunable coupling in circuit quantum electrodynamics using a superconducting charge qubit with a V-shaped energy level diagram. *Phys. Rev. Lett.* **106**, 083601 (2011).

[3] Motzoi, F., Gambetta, J. M., Rebentrost, P. & Wilhelm, F. K. Simple pulses for elimination of leakage in weakly nonlinear qubits. *Phys. Rev. Lett.* **103**, 110501 (2009).

[4] Bianchetti, R. *et al.* Dynamics of dispersive single-qubit readout in circuit quantum electrodynamics. *Phys. Rev. A* **80**, 043840 (2009).

[5] Eichler, C., Salathe, Y., Mlynek, J., Schmidt, S. & Wallraff, A. Quantum-limited amplification and entanglement in coupled nonlinear resonators. *Phys. Rev. Lett.* **113**, 110502 (2014).

[6] Chambers, J. M., Cleveland, W. S., Kleiner, B. & Tukey, P. A. *Graphical methods for data analysis*, (Wadsworth & Brooks/Cole, 1983).

[7] Burnham, K. & Anderson, D. *Model Selection and Multimodel Inference: A Practical Information-Theoretic Approach*, (Springer, New York, 2002), 2nd edn.
